# The Versatile Gasdermin Family: Their Function and Roles in Diseases

**DOI:** 10.3389/fimmu.2021.751533

**Published:** 2021-11-11

**Authors:** Ju Zou, Yixiang Zheng, Yan Huang, Daolin Tang, Rui Kang, Ruochan Chen

**Affiliations:** ^1^ Department of Infectious Diseases, Xiangya Hospital, Central South University, Changsha, China; ^2^ Hunan Key Laboratory of Viral Hepatitis, Xiangya Hospital, Central South University, Changsha, China; ^3^ Department of Surgery, UT Southwestern Medical Center, Dallas, TX, United States

**Keywords:** gasdermin, family gene, function, structure, GSDMD

## Abstract

The gasdermin (GSDM) family, a novel group of structure-related proteins, consists of GSDMA, GSDMB, GSDMC, GSDMD, GSDME/DNFA5, and PVJK/GSDMF. GSDMs possess a C-terminal repressor domain, cytotoxic N-terminal domain, and flexible linker domain (except for GSDMF). The GSDM-NT domain can be cleaved and released to form large oligomeric pores in the membrane that facilitate pyroptosis. The emerging roles of GSDMs include the regulation of various physiological and pathological processes, such as cell differentiation, coagulation, inflammation, and tumorigenesis. Here, we introduce the basic structure, activation, and expression patterns of GSDMs, summarize their biological and pathological functions, and explore their regulatory mechanisms in health and disease. This review provides a reference for the development of GSDM-targeted drugs to treat various inflammatory and tissue damage-related conditions.

## Introduction and Historical Background

Gasdermins (*GSDM*) form a gene family with similar structures and include the *GSDM* family of genes and *GSDM*-related genes. *GSDM*s were named based on the terms, “gastro” and “dermato,” as *GSDM* was found to be predominantly expressed in the gastrointestinal tract and skin. *GSDM* was first identified on chromosome 11 in a mouse model.

**Figure 1:**
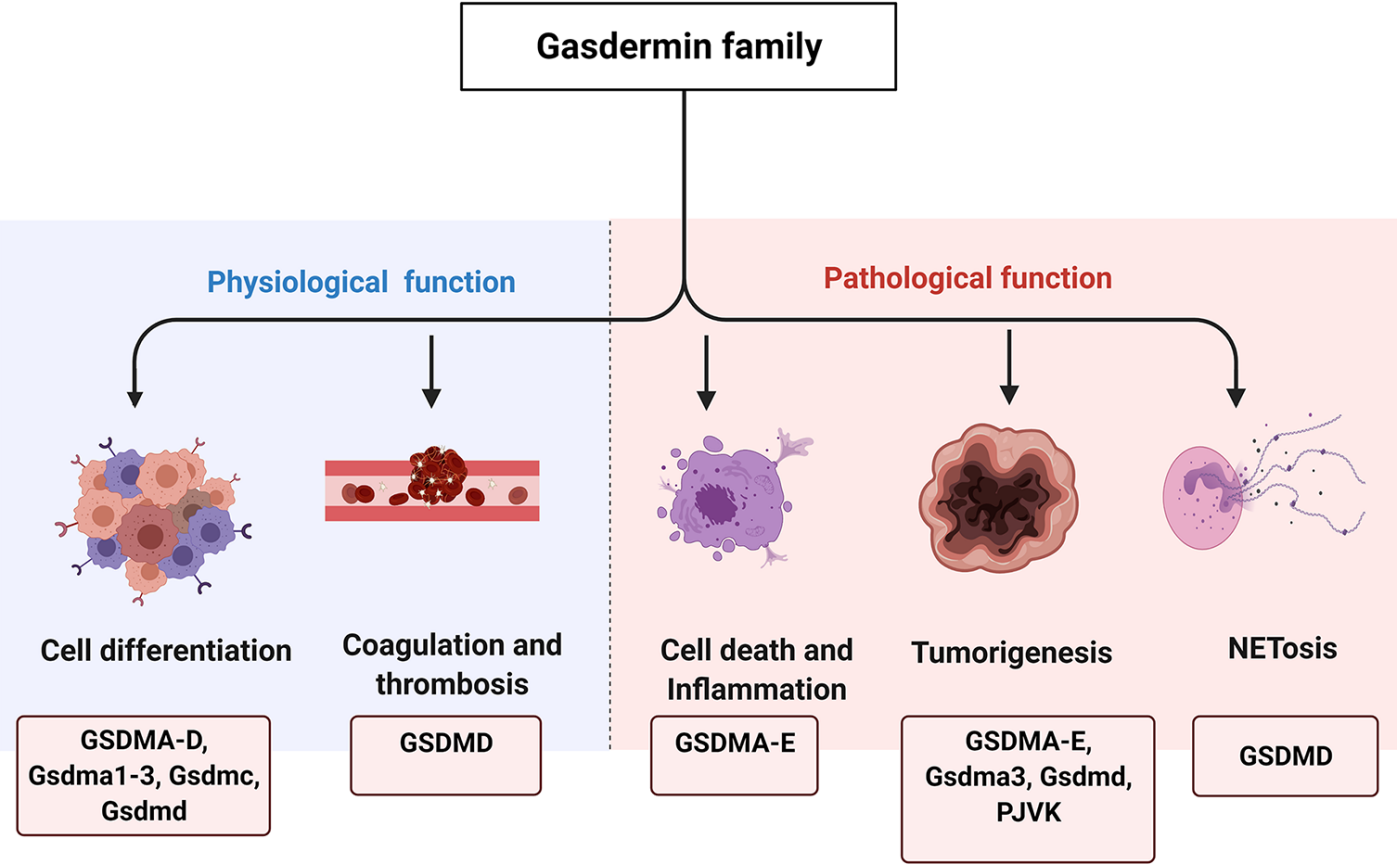
The physiological and pathological functions of GSDM family. GSDM family members are involved in various physiological and pathological processes, such as cell differentiation, coagulation and thrombosis, cell death and inflammation, tumorigenesis as well as NETosis.

Members of the *GSDM* family are widely expressed and may exert tissue-specific effects (**Table 1**), but their content-dependent functions remain largely unknown. For more than 15 years, scientists have been attempting to determine the structures and functions of GSDM family members, with some progress reported to date. Several lines of evidence suggest that the GSDM family plays diverse roles in biological and pathological processes, including in cell differentiation, cell proliferation, cell death, mitochondrial homeostasis, anti-microorganism, inflammation, and tumorigenesis (**Figure 1**). The direct role of these proteins in facilitating pyroptosis has also been intensively studied in recent years. In this review, we summarize current studies on the GSDM family structure and function and provide perspective on potential therapeutic targets for inflammatory and tissue damage-related conditions.

**Table 1:** GSDM family members, structures, functions, and disease relevance.

	GSDMA	GSDMB	GSDMC	GSDMD	GSDME	PJVK	Refs
**Aliases**	GSDM1/FKSG9	GSDML	MLZE	GSDMDC1/DFNA5L/FKSG10	DFNA5/ICERE-1	DFNB59/GSDMF	
**Gene and chromosomal location**	Human: *GSDMA: chr17q21* Mouse: *Gadma1* *Gadma2* *Gadma3* :*chr11D*	Human: *GSDMB:* *chr17q21* Not identified in mouse	Human: *GSDMC*: *chr8q24.2* Mouse: *Gsdmc1* *Gsdmc2* *Gsdmc3* *Gsdmc4* *:chr15D1*	Human: *GSDMD*: *chr8q24.3* Mouse: *Gsdmd* *:chr15D3*	Human: *GSDME*: *chr7p15.3* Mouse: *Gsdme/Dfna5:chr6B2.3*	Human:*PJVK*: *chr2q31.2* Mouse: *Pjvk/Dfnb59:chr2.3*	(1, 2)
**Domains**	GSDM-NT and GSDM-CT	GSDM-NT and GSDM-CT	GSDM-NT and GSDM-CT	GSDM-NT and GSDM-CT	GSDM-NT and GSDM-CT	GSDM-NT and zinc-finger	(3)
**Activating enzyme**	not known	Human: caspase 1 granzyme A	caspase-8	Mouse: caspase 1/11 Human: caspase 1/4/5 caspase 8 cathepsin G neutrophil elastase 3C protease of Enterovirus 71 (EV71)	caspase 3 caspase-8, caspase-7 granzyme B	not known	(1, 4–9)
**Lipid binding site**	GSDM-NT	full-length and GSDM-NT	GSDM-NT	GSDM-NT	GSDM-NT	GSDM-NT	
**Type of lipid**	phosphoinositides, cardiolipin, phosphatidic acid, phospatidylserine	phosphoinositides, phosphatidic acid, phosphatidylglycerol sulfatid	not known	phosphoinositides, cardiolipin, phosphatidic acid	phosphoinositides, cardiolipin, phosphatidylserin	not known	(4, 10–12)
**Tissue/cell-specific** **expression in human**	skin, tongue, esophagus, stomach, mammary glands, bladder, umbilical cord and T lymphocytes	airway epithelium, gastrointestinal tract, brain, endocrine tissue, bone marrow tissue, lung, liver, kidney, testis and lymphocytes	cerebral cortex, endocrine tissues, skin, trachea, spleen, esophagus, stomach, intestines, vagina and bladder	almost all human organs and tissue, including different subsets of leukocytes	brain, endocrine tissue, muscle tissue, gastrointestinal tract, endometrium and placenta	inner ear , neurons of the auditory system and testis	(1, 13–21)
**Biological function**	mitochondrial homeostasis	pyroptosis, anti-tumor immunity	not known	inflammation, pyroptosis, cytokine release, NETosis, bacteria killing	pyroptosis, anti-tumor immunity	not known	(17, 22–24)
**Associated disease**	alopecia, asthma, limited cutaneous system sclerosis,and inflammatory bowel disease	breast cancer and asthma	melanoma	atherosclerosis, type 2 diabetes mellitus, bullous pemphigoid, and cryopyrin-associated periodic syndromes sepsis and septic shock	deafness, cancer	deafness	(17, 22, 25–41)

GSDM family genes currently consist of GSDMA, GSDMB, GSDMC, GSDMD, GSDME (DNFA5) and PVJK (GSDMF). Every GSDM member has a distinct and restricted pattern of expression in different tissues leading to diverse diseases.

## GSDM Gene Family in Humans and Mice

### History and Nomenclature

In 1998, Van Laer et al. (42) conducted genetic analysis and position cloning to identify an insertion/deletion mutation in deafness autosomal dominant 5 (*DFNA5*). This gene was associated with non-syndromic hearing impairment in a Dutch family. In addition, the biological information center in Germany used the web-based tool Simple Modular Architecture Research Tool (SMART) to search and analyze a group of proteins with structures similar to that of *DFNA5*. They identified a new structure region in these proteins and named it as the *DFNA5* domain (43). In 2000, Japanese scientists (44) aiming to identify the causative genes of recombination-induced mutation 3 (*Rim3*) mutation in mice cloned a mice gene consisting of the *Dfna5* domain. *Rim3* mutant mice were characterized by epidermal abnormalities, such as thickened epidermis, loss of hair follicles, and stratified prickle-cell layers (44). Co-hybridized DNA technology was then utilized to isolate a novel mouse gene sequence, which was cloned from a mouse skin gene library; this mouse gene was named “*Gsdm*,” a word derived from “gastro” and “dermatome,” as it was predominantly expressed in the stomach and epidermis. Further, Southern blotting revealed the presence of *Gsdm* in the human genome (44). This prompted an update of the Simple Modular Architecture Research Tool website and renaming of DNFA5 to GSDM (Sanger Center, Pfams/getas PF04598; URL: http://www.sanger.ac.uk/cgi-bin/Pfam/getacc% 3FPF04598). Several other GSDM family members and GSDM-related proteins with homologous structures were identified. Currently, the GSDM family consists of four human genes (gasdemin A [*GSDMA*], gasdemin B [*GSDMB*], gasdemin C [*GSDMC*], and gasdemin D [*GSDMD*]) and eight mouse genes (*Gsdma1*, *Gsdma2*, *Gsdma3*, *Gsdmc1*, *Gsdmc2*, *Gsdmc3*, *Gsdmc4*, and *Gsdmd*) (**Table 1**). *GSDMB* is the most divergent member of the *GSDM* family. Although it is not present in the mouse and rat genomes, felines (cats and tigers) and canines (dogs) contain a *Gsdmb* orthologue (45). As shown in the study, human GSDMB is cleaved by chloroquine down-regulate caspase-1 (CASP1) and granzyme A at D236 and K244, respectively, whereas dogs, cats and tigers do not possess these key residues. This lack of activation sites in GSDMB in felines and canines indicate that they are deficient in the GSDMB-dependent pyroptosis pathway. Therefore, *GSDMB* is unique to the human genome to some extent. Moreover, two *GSDM*-related genes are common to both humans and mice: *GSDME*, also known as *DFNA5*, and *pejvakin* (*PJVK*), also known as *DFNB59* or *GSDMF*. Based on the divergent expression pattern and mutant-associated phenotypes compared to other *GSDM* family members, *GSDME* and *PJVK* were excluded from the *GSDM* family in some studies, although they have structures similar to those of the members in the family (46). Additionally, *PJVK* shows high similarity to *GSDME*. The nomenclature has been simplified to include all six members from *GSDMA* to *GSDMF* in a single *GSDM* family (4, 47).

### Protein Structural Basis of Autoinhibition and Activation

**Figure 2:**
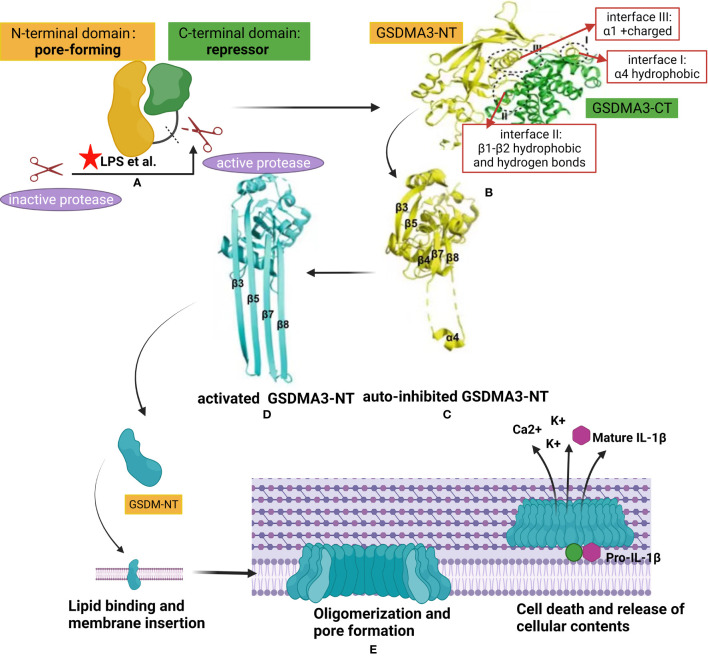
Structural auto-inhibition in GSDM family and mechanism of gasdermin membrane insertion and pore formation. **(A)** Interdomain interaction between the N-terminal of gasdermin (GSDM-NT) and C- terminal of gasdermin (GSDM-CT), with pore-forming and auto inhibitory characteristics respectively, keeps the full length GSDM protein in an auto-inhibited state. Different proteases, such as inflammatory caspases or granzymes, are activated by danger signals from pathogens like lipopolysaccharide (LPS). The latter was represented by a red pentacle in the image. The linker region of GSDMs is cleaved by the active protease releasing the GSDM-NT from the GSDM-CT. **(B)** (48) X-ray crystal structure of full-length GSDM and the interfaces mediating the inter-domain interactions (I-III) by forming electrostatic, hydrophobic, and hydrogen bonding. The GSDM-NT and GSDM-CT domains are colored yellow and green, respectively. **(C, D)** (49) Crystal structure of GSDMA3-NT in auto-inhibited conformation **(C)** and the pore conformation **(D)**. **(E)** A proposed universal model showing the pore formation by GSDM family. Once released from the GSDM-CT, the GSDM-NT is recruited to insert in the cell membrane by binding with lipid. Upon membrane binding, the GSDM-N concentrates and starts the oligomerization process forming pores leading to release of cellular contents,including ion flux and mature IL-1β, and finally cell death.

According to functional analysis, the GSDM protein, which contains around 220–480 amino acids, is the most important structure domain of GSDMs (43). Most protein products of GSDM family genes, except for PJVK/GSDMF, have three domains: the GSDM N-terminal (GSDM-NT) domain, linker region, and GSDM C-terminal (GSDM-CT) domain (**Figure 2A**). Both the N- and C-terminal domains are relatively well-conserved in the family, whereas the linker region is unique to each group (46). Notably, GSDM-NT of PJVK/GSDMF is directly followed by a small C- terminal domain that contains a zinc-finger domain with unknown function, suggesting evolutionary and mechanistic divergence within the GSDM family (3).

Given the high homogeneity among all GSDM-NT domains, most GSDMs may share a common mechanism of autoinhibition and activation to form pores in the membrane. Structurally, the GSDM-NT domain predominantly comprises β-strands with several α-helices (3). In contrast, GSDM-CT consists of α-helices and folds almost exclusively that form a compact globular conformation (50). The α1 and α4 helices of GSDM-NT, which play important roles in lipid binding and membrane insertion, can fold back on the concave side of GSDM-CT by forming electrostatic, hydrophobic, and hydrogen bonding. For example, Ding et al. reported that the crystal structure of full-length mouse GSDMA3 revealed three main interacting interfaces between the C- and N-terminal domains (3). First, the GSDMA3-CT domain forms a nonpolar surface that interacts with the hydrophobic region on the α4 helix of the GSDMA3-NT domain (**Figure 2B**, interface I). Second, the GSDMA3-CT domain forms another hydrophobic pocket that interacts with some hydrophobic residues on the β1–β2 loop in the GSDMA3-NT domain. The β1–β2 loop forms four hydrogen bonds with the C-terminal domain (**Figure 2B**, interface II). Third, the α1 helix in the GSDMA3-NT domain also provides a surface *via* predominantly charge-charge interactions with some residues in the GSDMA3–CT domain (**Figure 2B**, interface III). Therefore, inter-domain interactions between the GSDM-NT and GSDM-CT domains maintain the full-length GSDM protein in an auto-inhibited state (49).

In response to activation signals, GSDMs are cleaved in the central linker region by different inflammatory caspases or granzymes, as shown in the **Table 1**, to generate a 31-kDa GSDM-NT fragment and 22-kDa GSDM-CT fragment (3, 51). GSDM-CT typically functions as a repressor when binding to GSDM-NT; thus, its overexpression inhibits cell death (5). GSDM-NT, when uninhibited by GSDM-CT, binds to membrane lipids and extensively form pores, resulting in cytotoxicity and cell death (3, 13, 51). For GSDMD protein, inflammatory caspases are activated through intracellular lipopolysaccharide (LPS) binding (caspases-11, -4, and -5) or the inflammasome (caspase-1) (**Figure 2A**). Upon activation, inflammatory caspases cleave the linker region and release the N-terminal domain from the autoinhibitory C-terminal domain, which binds to lipids in the membrane and forms a pore.

The molecular mechanism of pore formation by GSDMs has been confirmed previously (3, 10, 52–54). Structurally, the entire β3–β4–β5 region in the auto-inhibited stated GSDMA3-NT is extended to form the first transmembrane β-hairpin, with β7, β8, and α4 binding the C-terminal domain form the second β-hairpin (**Figure 2C**). Once the balance of auto-inhibition is disrupted through mechanisms such as cleavage of GSDMD by mammalian caspase 1, mouse caspase 11, or human caspase 4/5, GSDMA3-NT is released from GSDMA3-CT and undergoes conformational changes. Each GSDMA3-NT domain contributes four extended β-strands to pore formation (**Figure 2D**). The hydrophilic and hydrophobic residues of the four β-strands are alternately arranged, with hydrophobic residues oriented outward to contact the surrounding lipid (3, 49).

The conformational changes leading to pore formation are triggered by binding of the N-terminal domain to membrane phospholipids, with a positively charged pocket and negatively charged group, respectively. The surface of this pocket is fully masked by the GSDM-CT domain in the auto-inhibited full-length of GSDMs (3). GSDMD-NT was reported to tightly bind acidic phospholipids, such as phosphoinositides and cardiolipin, but weakly bind phosphatidic acid and phosphatidylserine, reducing the stability of the target cell membrane (3, 10). The lipid-binding properties of the other GSDMs-NT domain is similar to that of GSDMD, suggesting a common membrane-targeting mechanism in the GSDM family. Notably, the function of the GSDM pore can differ depending upon the target cell type, level of GSDM protein expression, timing of activation, and efficiency of interaction. For example, LPS can elicit GSDMD-dependent release of mature interleukin (IL)-1β from live human monocytes (55). Furthermore, ion fluxes though GSDMs pores have a profound impact on cellular signal pathways because these pores act as large and non-selective membrane channels (**Figure 2E**).

Interestingly, in the absence of environmental lipids, GSDM-NT and GSDM-CT are connected despite cleavage of the linker region (3). This finding indicates that lipids are required to dissociate GSDM-NT from GSDM-CT. In contrast, GSDM-CT mutations in neighboring residues of GSDM-NT enable full-length GSDMs to form pores, supporting the hypothesis that GSDM-NT and GSDM-CT dissociation is not necessary for pore formation (3). Moreover, full-length GSDMB displays a similar lipid-binding ability, in contrast to the GSDMB-NT domain alone, suggesting that the GSDMB-CT domain does not impair the lipid binding of GSDMB (56). In summary, the precise molecular mechanisms underlying the GSDM-NT/CT interaction and GSMD protein modification and activation require further clarification.

## Expression Pattern of GSDM Family Genes in Normal Tissue

Every GSDM member has a distinct and restricted pattern of expression in different tissues. We investigate the RNA and protein expression of GSDM family genes in human and murine tissues by reviewing previous publications and two public databases, the Human Atlas Protein (http://www.proteinatlas.org) and MGI-mouse Gene Expression Database (http://www.informatics.jax.org/expression.shtml). Human GSDMA is mainly expressed in epithelial cells of the skin, tongue, esophagus, stomach, mammary glands, bladder, and umbilical cord (**Table 1**) (14, 15). Furthermore, GSDMA protein was detected in T lymphocytes (16). Analysis using reverse transcription-polymerase chain reaction indicated that there is a more distinct tissue-specific expression of *Gsdma* in mice (46). *Gsdma1* is expressed in the suprabasal epidermis, cornea, hair follicles, and forestomach (13, 15, 44, 57). *Gsdma2* is mainly expressed in the stomach gland. *Gsdma3*, where *Rim3* mutation occurs, is primarily found in the sebaceous gland of the skin (58, 59).

GSDMB, also known as GSDML, is composed of 411 amino acids and is the most divergent member of the GSDMB family. *GSDMB* is exclusively expressed in humans but not in the mouse and rat genomes. However, a *Gsdmb* orthologue is reportedly expressed in rodents. The human GSDMB has six splicing variants; their expression is detected in numerous human tissues and cells, such as the airway epithelium, gastrointestinal tract (esophagus, stomach, small intestine, and colon), brain, endocrine tissue, bone marrow tissue, lung, liver, kidney, testis, and lymphocytes (**Table 1**) (1, 13, 14, 17, 18).

GSDMC, also known as melanoma-derived leucine zipper-containing extranuclear factor, was named after metastatic mice melanoma cells in which its increased expression was observed and the gene was first identified (60). Human GSDMC is expressed in the cerebral cortex (61), endocrine tissues, skin, trachea, spleen, esophagus, stomach, intestines, vagina, and bladder (**Table 1**) (18). There are four *Gsdmc* orthologous genes in mice (46), namely, mouse GSDMC1, GSDMC2, and GSDMC4. These orthologous genes are expressed in the stomach, large and small intestines, bladder, and prostate, whereas GSDMC3 expression is restricted to the bladder, prostate, and large intestines (J:171409 GUDMAP Consortium, http://www.gudmap.org. 2004).

GSDMD is best known for its role in mediating pore formation and subsequent pyroptosis. Human *DFNA5L/GSDMD*, mouse *Dfna5l/Gsdmd*, and rat *Dfna5l/Gsdmd* were first identified and characterized *via* bioinformatics analysis of genomic data (61). Almost all human organs and tissues, including different subsets of leukocytes, express GSDMD mRNA and proteins (**Table 1**) (16, 18). GSDMD is also widely expressed in the stomach, gut, colon, heart, spleen, liver, lung, and urinary system of mice (62, 63).

The expression patterns of two *GSDM*-related genes, *GSDME* and *GSDMF*, were investigated in human and mice. The mRNA and protein expression of *GSDME* was detected in a wide range of human tissues and cells, such as the brain, endocrine tissues, muscle tissues, gastrointestinal tract, endometrium, and placenta (**Table 1**). Mouse GSDME is expressed in the gastrointestinal tract, ears, heart, adipose tissues, endocrine glands, spleen, mammary glands, liver, and lungs (19, 64). GSDME is also expressed in different species of lower vertebrates. For instance, two *GSDME* orthologous genes, *GsdmEa* and *GsdmEb*, were identified in bony fish (56).

PJVK (GSDMF) consists of 352 amino acids; its gene is on chromosome 2q31.1-q31.3. This gene has been implicated in autosomal recessive deafness and has been detected in the inner ear and neurons of the auditory system (20). However, it was initially cloned from the human testis (**Table 1**). Furthermore, *PJVK* orthologous genes are present in early chordates and invertebrates, suggesting that GSDM family members evolved from these organisms. Moreover, *PJVK* shares a high similarity with *GSDME*. In fact, the RNA expression of *PJVK* is most abundant in the testis, but it is also widely expressed in other tissues as revealed in various databases (such as Consensus, GTEx, and FANTOM5) (20, 65, 66).

Protein and RNA analyses revealed that *GSDM* family genes exhibit a unique tissue-specific expression pattern. This pattern appears to be conserved between human and mouse species. A systematic and comparative expression investigation and analysis of human and mouse should be conducted to clarify these patterns.

## Physiological and Pathological Function of GSDM Family Genes

### Cell Differentiation

Studies of the distinct expression patterns of *GSDM* family members in the gastrointestinal tissue revealed their potential roles in cell proliferation and differentiation (**Figure 1**) (67). In the esophageal and gastric epithelium, *GSDMA* is preferentially expressed in the differentiated pit layers, indicating a potential function in mature epithelial cells. *GSDMB* mRNA is predominantly expressed in the basal region of the esophagus and in the stomach, where stem cells are typically located. It is also detected in the isthmus or neck of the stomach, where rapidly proliferating precursors of pit cells are harbored. *GSDMC* is mainly expressed in the differentiated supero-basal region of the esophagus; however, it is not detected in any region of the gastric epithelium. *GSDMD* is expressed in the differentiating cells of the esophagus and differentiated and differentiating regions of the stomach. In the small intestines and colons of mice, *Gsdmc* and *Gsdmd* exhibit a distinct expression pattern, as determined using reverse transcription-polymerase chain reaction (46). These studies indicate that the GSDM family plays a critical role in cell differentiation based on the varying levels of GSDM proteins in epithelial cell in the gastrointestinal tract with various levels of differentiation. However, direct evidence of this role is required.

Mouse homologues of *GSDMA*, which include *Gsdma1*, *Gsdma2*, and *Gsdma3*, are clustered on chromosome 11 (46). *Gsdma3* is predominantly expressed in differentiated epidermal cells of the skin. Histological analysis of the *Gsdma3* mutation revealed epidermal cell hyperplasia in the upper hair follicles and an abnormal anagen phase at the first hair cycle. Furthermore, the results of immunohistochemical analysis showed that hyperproliferation and misdifferentiation occurred in the upper follicular epidermis among *Gsdma3* mutants (58). These results suggest that *Gsdma3* is involved in the proliferation and differentiation of epidermal stem cells. Moreover, hair follicle differentiation defects, such as flattened Cuh, reduced keratin levels in the cortex, and abnormal arrangement of trichohyalin in the inner root sheath, have been observed in *Gsdma3* mutant mice with alopecia and excoriation. Furthermore, Gsdma3 and Msx2 are co-expressed in the matrix and inner root sheath, and overexpression of Gsdma3 in the mouse skin promotes activation of Msx2 pathway *in vivo* (68). In summary, GSDMA3 protein is involved in the Msx2/Foxn1/acidic hair keratin pathway, whereas increased apoptosis is observed in the hair follicles of mice with mutated Gsdma3. These findings suggest that GSDMA3 plays a crucial role in normal hair follicle differentiation (68).

### Coagulation and Thrombosis

The development of blood clots and thrombosis is often implicated in various physiological and pathological processes. In response to the presence of LPS or a specific PAMP from *Escherichia coli*, such as the type III secretion system inner rod protein EprJ, the activation of canonical/non canonical inflammasome mediates GSDMD activation, pyroptosis, and tissue factor release in primary bone marrow-derived macrophages, resulting in systemic coagulation and thrombosis in C57BL/6J mice (**Figure 1**) (69). Although bacterial infection can induce inflammasome activation and pyroptosis in macrophages, the underlying mechanisms remain unclear. Recently, a study (70) showed that Ca^2+^ influx, which was mediated by the GSDMD pore, resulted in phosphatidylserine exposure to peripheral leukocytes and splenocytes, thereby leading to life-threatening disseminated intravascular coagulation and endotoxemia in mice. Phosphatidylserine exposure and tissue factor activity was attenuated in murine macrophages cultured with low Ca^2+^-containing medium. Casp11 deletion, Gsdmd ablation, or phosphatidylserine or tissue factor neutralization significantly prevented LPS-induced disseminated intravascular coagulation in murine models with endotoxemia (70). In the clinical setting, an increase in the biomarkers of GSDMD activation, which include the plasma levels of IL-1A and IL-1B, was significantly correlated with the formation of thrombin-antithrombin complex, a high disseminated intravascular coagulation score, and phosphatidylserine externalization in peripheral leukocytes of septic patients (70). This inflammasome-related coagulation in macrophages is further enhanced by activation of the stimulator of interferon genes pathway (71). Whether other members of GSDMs are involved in coagulation and thrombosis and how they interact with initiators of coagulation require further investigation.

### Tumorigenesis

Tumorigenesis is associated with alterations in various cellular processes. GSDMA shows enhanced apoptosis-inducing activity in human gastric pit cells, and its expression and function are regulated by the TGF-β/transcription factor limdomain only 1 signaling pathway (**Figure 1**). This pathway is associated with the apoptosis of gastric pit cells, as GSDMA is frequently suppressed in esophageal and gastric cancers (57).

Moreover, stable transfection of GSDMB promotes cell proliferation of the ovarian cell line CHO-K1, whereas genetic depletion of GSDMB inhibits the growth of HeLa cells (**Figure 1**) (72). Consistently, GSDMB is significantly more upregulated in in uterine cervix cancer tissues compared with other precancerous tissues. Additionally, GSDMB is expressed in all examined cases of esophageal and gastric cancers (67). GSDMB is significantly more upregulated in breast carcinoma compared to in normal breast tissue. Moreover, both isoforms of GSDMB, GSDMB1 and GSDMB2, promote invasion and metastasis of the MCF7 breast carcinoma cell line *in vitro*, whereas silencing of these isoforms in the HCC1954 breast carcinoma cell line significantly decreased migration and invasion (22). *In vitro* data from MCF7 cells and *in vivo* data from an intracardiac experimental metastatic mice model suggested that GSDMB interacts with several molecules and signaling pathways, such as Hsp90, Rac-1, and Cdc-42 Rho-GTPases, to induce tumorigenesis (22). *GSDMB*, which is in amplicons and genomic regions, is often amplified during tumorigenesis. Thus, GSDMB may play a role in tumor progression and metastasis. However, the specific functions of GSDMB in carcinogenesis, cancer metastasis, and progression are unclear.

GSDMC, also known as melanoma-derived leucine zipper-containing extranuclear factor, was originally isolated from mouse melanoma cells and obtained from the mouse melanoma cell B16-BL6 cDNA library (60). The expression levels of mouse GSDMC were positively correlated with the metastatic ability of B16 melanoma cell lines (60). However, it is unclear whether GSDMC is involved in one or more processes in the invasion-metastasis cascade. In addition, whether upregulated GSDMC expression upon acquisition of metastatic potential in melanoma cells is only an accompanying phenomenon is unclear. Nevertheless, knockdown of GSDMC attenuated the proliferation of colorectal cancer cell lines, such as DLD-1 and LoVo cells, indicating a pro-tumorigenic role (73). Furthermore, GSDMC shRNA resulted in a significant decrease in xenograft tumor growth in mice (73). Consistent with the above findings, GSDMC expression was suppressed in several cases of esophageal squamous cell carcinoma, thus supporting that GSDMC is a tumor-suppressor gene (**Figure 1**) (67). However, it is unknown whether GSMDC acts as a tumor-promoting regulator or an anti-tumor regulator in cancer development; thus, further investigation is required.

In addition to GSDMA, GSDMB, and GSDMC, GSDMD reportedly inhibited proliferation of the gastric cancer cell line MKN28 in a colony formation assay (67). However, a significantly upregulated GSDMD was observed in various types of cancer, such as cervical cancer, liver cancer, ovarian cancer, and pancreatic cancer. The results of these expression assays implicate a role for GSDMD in promoting carcinogenesis. Moreover, GSDMD expression is correlated with CD8^+^ T cell markers in primary tumors of lung adenocarcinoma, lung squamous cell carcinoma, and melanoma cohorts of The Cancer Genome Atlas (74). The level of GSDMD cleavage was increased in human activated CD8^+^ T cells, whereas GSDMD gene depletion decreased the cytolytic capacity of CD8^+^ T cells; thus, GSDMD was required to facilitate an optimal cytotoxic T-lymphocyte response to lung cancer cells (74). Inhibition of GSDMD also attenuated tumor proliferation by promoting intrinsic mitochondrial apoptosis *via* the EGFR/Akt signaling pathways in non-small cell lung cancer cell lines (PC9 and H1703) (75).

Although the expression and post-translational modification of GSDME in various types of cancer have been examined in several studies, the exact role and underlying molecular mechanisms of these processes remain ambiguous (76, 77). GSDME may be a tumor suppressor, as its expression is decreased in breast, gastric, and colorectal cancers (78–80). Furthermore, GSDME deficiency accelerates the proliferation of gastric, colorectal, and melanoma cancer cell lines (11). Upregulation of GSDME also increased the drug susceptibility of a chemotherapy-resistant melanoma cell line by upregulating caspase-3, which mediated pyroptosis (4).

### Cell Death and Inflammation

**Figure 3:**
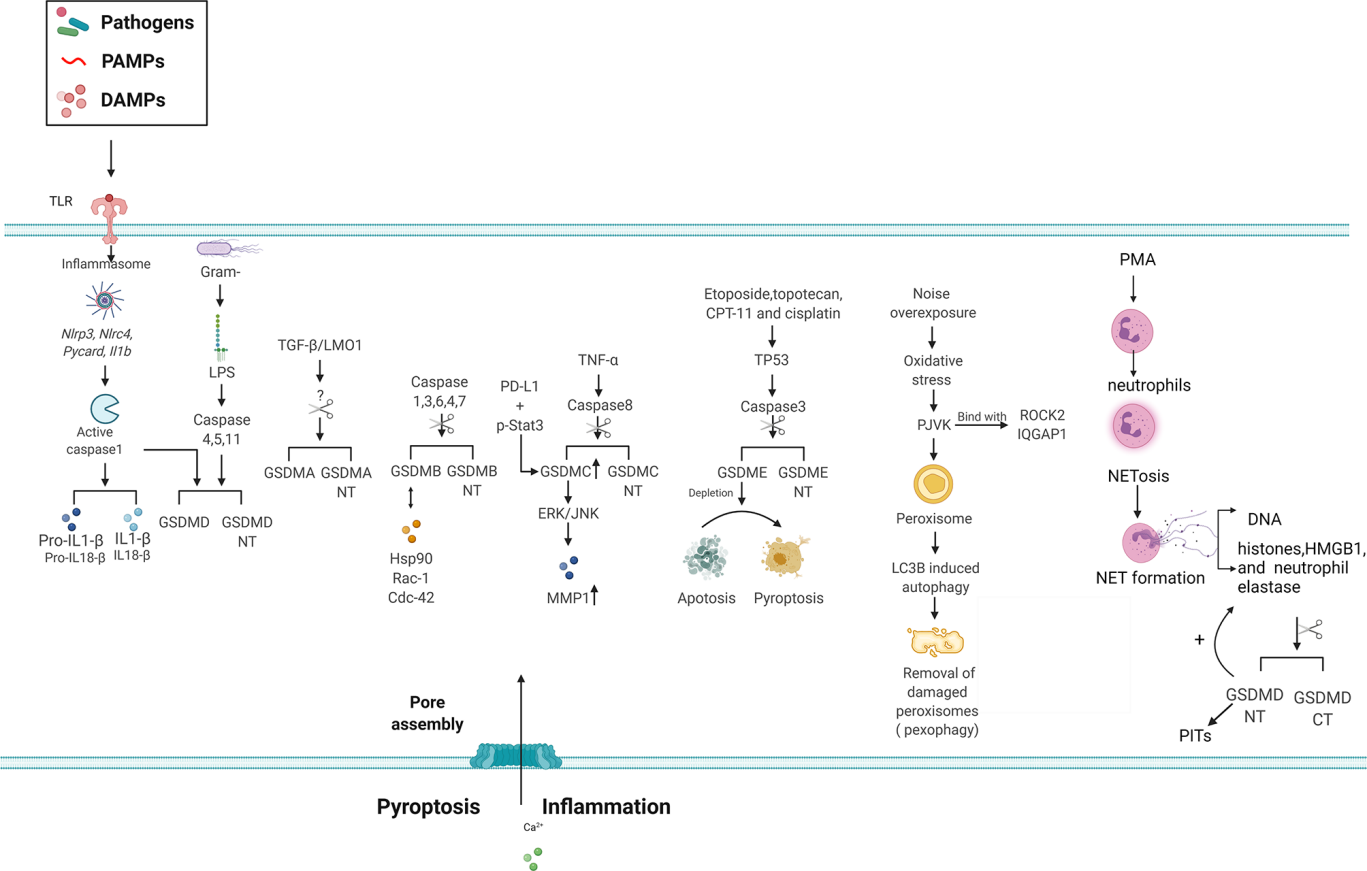
Signaling pathways of GSDM family members in inflammasome signaling, pore formation and cell death. GSDMD is involved in pyroptosis *via* canonical and non-canonical inflammasome mediated pathways following cleavage by inflammatory caspases. The N-terminal domain of GSDMA-E can bind with membrane lipids of the plasma membrane and form pores, allowing release of the inflammatory cytokines IL-1BIL-1B and IL-18 and induction of pyroptosis. However, the inflammatory caspase responsible for GSDMA cleavage remain elusive. Various stimuli and upstream signals like PD-L1, TP53 and TNF-α, can lead to GSDMs cleavage and subsequent cell death. GSDMs are able to bind with other molecules, such as Hsp90, Rac-1 and ROCK2, and the following function require further investigation. Alternatively, depletion of GSDME can switch apoptosis to pyroptosis in cells. The ability of PJVK to bind membrane lipids or form pores in the plasma membrane is uncertain, however it is implicated autophagy related pexophagy. In addition, GSDMD leads NETosis as the key effector GSDMD is cleaved by neutrophil elastase during NETosis induced by classic stimulants such as PMA, releasing the active GSDMD-N.GSDMD-N, Gasdermin N-terminal; GSDMA, Gasdermin A; GSDMB, Gasdermin B; GSDMC, Gasdermin C; GSDMD, Gasdermin D; GSDME, Gasdermin E; HSP90, heat shock protein 90; IL-1BIL-1B, interleukin-1β; IL-18, interleukin-18; PJVK, Pejvakin; PD-L1, programmed death-ligand1; ROCK2, rho associated coiled-coil containing protein kinase 2; Rac-1, ras-related C3 botulinum toxin substrate 1; Cdc42, cell division cycle 42; IQGAP1, IQ Motif Containing GTPase Activating Protein 1; MMP1, Matrix Metallopeptidase 1; ERK, extracellular regulated protein kinase; JNK,c-Jun N-terminal kinase; TGF-β, transforming growth factor-β; LOM1, LIM Domain Only 1; PMA,phorbol 12-myristate 13-acetate; Netosis, neutrophil extracellular trap formation (NETosis); PITs: pore induced intracellular pits.

Several studies have shown that GSDMs induce cell death and inflammation (**Figure 1**). Particularly, the role of GSDMD in pyroptosis has been widely studied. Pyroptosis is a lytic form of cell death driven by inflammatory caspases and characterized by cell swelling, nuclear condensation, cell membrane disruption, and inflammatory cytokine and DAMP release (81, 82). Inflammatory caspases orchestrate the formation of a multi-protein platform known as the inflammasome, thereby inducing the cleavage and release of proinflammatory cytokines, such as IL-1B and IL-18, from macrophages (83) (**Figure 3**). In addition to canonical inflammasome activation, a signaling pathway of noncanonical inflammasome regulated by caspase-11/4/5 was identified (25). During infection with gram-negative bacteria, LPS in the outer membrane of the bacteria is imported into the intracellular space of the host cell. It then directly binds to and activates caspase-11 in mice and caspase-4 and -5 in humans (84–87). However, the mechanism underlying the inflammatory caspases’ modulation of the intracellular physiological events remains unclear.

Three groups (5, 6, 88) demonstrated that GSDMD is a fundamental substrate of caspase-1, -11, -4, and -5. A previous study (5) employed genome-wide clustered regularly interspaced palindromic repeat/Cas9 technology and siRNA-mediated knockdown techniques to detect caspase-11- and caspase-1-mediated pyroptosis in the bone marrow macrophages of mice. The researchers identified GSDMD as the major mediator of cytoplasmic LPS-mediated pyroptosis in the macrophages of humans and mice. This finding was supported by another study (6), which employed a forward genetic approach to screen ethyl-N-nitrosourea-mutagenized mice for mutations that diminished induction of the caspase-11-mediated noncanonical signaling pathway. This screening approach also linked GSDMD to intracellular LPS-induced pyroptosis and IL-1B maturation and release (6). In addition, another study (88) identified GSDMD as a molecule that directly interacted with inflammasomes. This was confirmed by analysis of LPS-stimulated macrophage RAW264.7 cell lines and primary cells using quantitative mass spectrometry technology. Apoptosis was shown to be suppressed by pyroptosis in response to LPS and nigericin or to *Salmonella typhimurium* treatment. These results suggest an interplay between apoptosis and pyroptosis (88). These studies suggest also that GSDMD is a direct substrate of inflammatory caspases (caspase-1, -11, -4, -5) and the executioner of inflammatory cytokine release and pyroptosis, as well as clarify the detailed mechanism of how GSDMD mediates noncanonical inflammasome pathways and pyroptosis. GSDMD is also cleaved by caspase-1, indicating a role for this substrate in caspase-11, -4, and -5-independent canonical inflammasome activation and pyroptosis (89). The results of a study using genome-wide clustered regularly interspaced palindromic repeat/Cas9 involving the human monocyte cell line and ethyl-N-nitrosourea-mutagenized mice showed that the expression and function of GSDMD was positively regulated by the interplay between transcription factors and interferon regulatory factor-1 and -2 (90, 91). In contrast to GSDMD, other members of the GSDM family, such as GSDMA, GSDMB, and GSDMC, are not cleaved by caspase-1 and caspase-11. This may be because of the lack of the _272_FLTD_275_ motif in GSDMD (5). However, overexpression of the gasdermin-N domain of GSDMA3 leads to induction of significant pyroptosis in 293T cells. This suggests that GSDMs are pyroptosis-inducing proteins activated by autoinhibition of their N-terminal domain.

The precise mode of the execution of cell death by the GSDMD N-terminal has been explored. Caspase-matured GSDMD-NT is recruited to bind to the inner lipid membrane and penetrate it by forming pores as the key effector mechanism of pyroptotic cell death (3, 54, 92). Using a cell-free method, the cleaved and recombinant GSDMD-NT were found to bind to liposomes *in vitro*, indicating that GSDMD mediates its own recruitment to membranes. GSDMD-NT is exclusively associated with lipids of the inner wall of the plasma membrane, explaining why GSDMD only lyses cells from the inside and prevents unwarranted cell death and tissue damage. In addition, GSDMD binds to cardiolipin, a component of prokaryotic and mitochondrial membranes, which may explain its proposed bactericidal activity. Indeed, the recombinant protein of GSDMD-NT but not the full-length protein and GSDMD-CT induces extensive killing of *Listeria monocytogenes*, *Staphylococcus aureus*, and *E. coli* (10). Interestingly, the GSDMD-NT-induced membrane pores also function as conduits for IL-1 family cytokine release and other cytosolic contents, including ions, from cells (93–96). In addition, inhibiting GSDMD-induced pyroptosis using chemicals such as disulfiram or dimethyl fumarate, is a promising strategy for reducing inflammation (97, 98). Furthermore, liquid chromatography-tandem mass spectrometry revealed that dimethyl fumarate and endogenous fumarate reacts with GSDMD at critical cysteine residues to form *S*-(2-succinyl)-cysteine. These critical cysteine residues include Cys^191^ in humans and Cys^192^ in mice. Succination of GSDMD prevents its interaction with caspase 1, which then limits the processing, oligomerization, and capacity to induce death of HEK293T cells (98).

In addition to its role in pyroptosis, GSDMD is a critical mediator of neutrophil extracellular trap (NET) formation and other modes of cell death (**Figures 1**, **3**). Particularly, NET formation is observed in neutrophils, in which neutrophil extracellular traps (NETs) are formed during this type of cell death (99–101). NETs are composed of DNA and non-chromatin molecules, such as histones, HMGB1, and granule proteins. Pore formation in neutrophils induced by GSDMD promotes cell rupture and NET extrusion into the extracellular space. Moreover, neutrophil elastase cleaves GSDMD to produce GSDMD-NT during NET formation. In turn, GSDMD-NT promotes protease activation and nuclear expansion in a feed-forward loop (101). Interestingly, GSDMD induces the formation of pore-induced intracellular pits, which triggers a multifaceted defense against intracellular bacteria by trapping pathogens within the cellular debris (102) (**Figure 3**). However, further investigation is required to clarify the antibacterial activity of endogenous GSDMD *in vivo* and *in vitro.*

Similar to GSDMD-NT, overexpression of GSDMA-NT or GSDMA3-NT in 293T cells may result in pore formation in the plasma membranes and the induction of pyroptosis-like features (3, 5). Upregulation of human GSDMA promotes TGF-β-dependent apoptosis in gastric epithelial cell lines (57), whereas mouse Gsdma3 has been implicated in the TNF-α-mediated apoptosis signal pathway in skin keratinocytes (103). The Gsdma3 Y344H mutant protein and Gsdma3-NT domain display similar pro-autophagic abilities, which is associated with mitochondria and generation of reactive oxygen species in HEK293T and HaCat cells and mouse models. Loss of the conserved self-regulation of Gsdma3 results in autophagy and cell death (51). However, protease cleavage in human and mouse GSDMA and their regulatory mechanisms are not well-understood.

Both the full-length and GSDMB-NT can bind to membrane lipids such as phosphoinositide and glycolipid sulfatide, distinguishing them from other GSDMs (104). GSDMB can be cleaved by caspase-1, -3, -4, -6, and -7 within the pore-performing domain at the 88DNVD91 or D236 sites, which is similar to cleavage of the pore-forming domain in GSDMD (1, 105, 106). However, GSDMB-NT but not the full GSDMB protein induces pyroptosis-like features in human HEK 293T cells (3). This suggests that binding of lipids of the full GSDMB protein leads to the production of GSDMB-NT. Artificially truncated GSDMC-NT also induces pyroptosis. Under hypoxic conditions, p-Stat3 physically interacts with PD-L1 and facilitates its nuclear translocation, which promotes GSDMC/caspase-8 activation, mediates the non-canonical pyroptosis pathway, and induces tumor necrosis in MDA-MB-231 cells (107). The N-terminal domain of GSDMC is sufficient to induce pyroptosis both *in vivo* and *in vitro*. However, future studies should focus on the signals that can activate GSDMB and GSDMC and the mechanism underlying cleavage of GSDMB and GSDMC, which mediate cell death.

Recent studies have indicated that GSDME induces tumor cell death by inducing apoptosis and pyroptosis (108–110), which may explain its potential tumor suppressive activity (111, 112). Mutations in intron 7 of *GSDME* cause sensorineural hearing loss because of skipping of exon 8 at the pre-mRNA level and translation of a C-terminally truncated protein (113). Although the full-length product does not have cytotoxic activity, its truncated form does (112). Moreover, GSDME expression can be induced by the transcription factor TP53 in response to etoposide, a potent inducer of apoptosis (114). The mRNA expression of *GSDME* is remarkably induced by gamma ray irradiation in the colon of TP53^(+/+)^ mice but not in that of TP53^(–/–)^ mice, suggesting that cooperation between TP53 and GSDME is required to mediate cell death. GSDME is a physiological substrate of caspase-3 when it is stimulated by apoptotic triggers such as etoposide or vesicular stomatitis viral infection. Mechanistically, GSDME is cleaved by caspase-3 at Asp270 to generate a necrotic GSDME-NT domain that translocates to the plasma membrane, where it increases cell membrane permeability. In addition, secondary necrosis and pyroptosis are induced in HeLa cells. Moreover, GSDME specifically requires caspase-3 but not caspase-7 to switch TNF-induced apoptosis to pyroptosis in HeLa cells. Compared to wild-type mice, *Gsdme* knockout mice are resistant to the toxic effects of chemotherapeutic drugs, such as cisplatin, 5-fluorouracil, and bleomycin (4). These findings indicate that GSDME is a central molecule that regulates apoptotic cell disassembly and progression to secondary necrosis. Furthermore, these findings reveal a molecular mechanism for secondary necrosis (47). Interestingly, it has been suggested that PJVK localizes to the membrane of peroxisomes in inner hair cells (115). Furthermore, it directly recruits the autophagy protein LC3B to induce autophagy-mediated removal of damaged peroxisomes (pexophagy) following oxidative stress caused by noise overexposure (116). PJVK-driven pexophagy is followed by peroxisome proliferation, which protects auditory hair cells from oxidative damage. Two proteins, rho-associated coiled-coil containing protein kinase 2 and the scaffold protein IQGAP1, were shown to bind the C-terminal region of PJVK in a cochlear cDNA library (117). Whether PJVK can be cleaved by a protease and form membrane pores requires further investigation.

## Implicated Diseases Associated With GSDM Family Genes

### Dermatological Disorders

Mutated *Gsdma3*, reduced Coat 2, res-denuded, and bareskin in mice have been identified in mice with alopecia (59, 114), revealing *Gsdma3* as a mutation hotspot. Spontaneous mouse mutant defolliculated and chemical induced mutant finnegan also harbor several gsdmd3 mutations. *Gsdm3* (defolliculated) is a B2 insertion next to the 3′ splice site of exon 7, whereas *Gsdm3* (finnegan) is a point mutation in T278P. Different *Gsdma3* mutations are associated with different skin-related phenotypes, including aberrant sebaceous gland differentiation, shortened hair shafts, altered catagen stage of the hair cycle, and loss of the hair follicle, which eventually lead to hair loss (**Table 1**) (26, 118). However, there are no visible developmental skin abnormalities in *Gsdma3* ^–/–^ mice (51), suggesting that these mutations have gain of function effects. GSDMC is also involved in the physiological and pathological processes of skin metabolism. In response to UV irradiation, GSDMC is upregulated in human keratinocytes. Moreover, GSDMC overexpression contributes to skin damage by promoting ERK-JNK-mediated MMP-1 upregulation in primary human skin keratinocytes (119).

### Cancer

The expression and function of GSDM family genes are associated with several types of tumors (**Table 1**). In this section, we focus on the clinical significance of GSDMs in different types of cancer and their correlation with metastasis, early diagnosis, response to treatment, and prognosis. GSDMA is frequently suppressed in human esophageal, gastric, and skin cancer tissues and tumor cell lines. Furthermore, it is associated with cell death and the sensitivity of cancer to treatment (67, 73). However, whether GSDMA is associated with cleavage and pore formation in cancer cells remains unknown.

In contrast, GSDMB overexpression is observed in many types of cancers, such as human gastric cancer, hepatocellular carcinoma, colon cancers, cervical cancer, breast cancer, and their corresponding cancer-derived cell lines (72). In patients with human epidermal growth factor receptor 2-positive breast cancer, increased GSDMB in tumor cells is associated with an increased incidence of metastasis, reduced survival, poor response to human epidermal growth factor receptor 2-targeted therapy, and poor prognosis. GSDMB is co- expressed with ERBB2 (22, 120) in cancer tissues. However, the mechanism underlying cell survival in cells with an upregulated GSDMB remains unknown. Nevertheless, GSDMB-NT induces pyroptosis when overexpressed in cultured cells.

Overexpressions of GSDMC and GSDMD have been observed in various types of cancer (60). However, there is no consensus on the function of GSDMC in cancer, as it exerts pro- and anti-tumor activity in different tumor types. Studies (56, 57, 79, 80) involving large, multicenter cohorts that examined different types of cancer revealed the role of GSDMC in cancer and confirmed the above findings. Chemotherapeutic reagents, such as etoposide, topotecan, CPT-11, and cisplatin, can induce pyroptosis in cancer cells *via* a GSDME-dependent mechanism, whereas they promote apoptosis in GSDME-negative cells (4). These studies suggest that GSDME is involved in tumorigenesis. The possible mechanism involves inactivation of GSDME *via* methylation, which leads to decreased apoptosis, contributing to carcinogenesis (121).

### Asthma

Asthma is a chronic airway inflammation of the lung tissues that are infiltrated with massive inflammatory cells. This complex clinical syndrome is characterized by increased airway hyperresponsiveness and reversible airway obstruction. Aside from environmental risk factors, up to 75% patients with asthma have a genetic susceptibility background (122). A study of Korean children (27) revealed that the susceptibility to asthma and intermediate asthma phenotypes, such as elevated IgE and bronchial hyperresponsiveness, is associated with GG of *GSDMA* (rs7212938) and TT of *GSDMB* (rs7216389) (27). The levels of DNA methylation and gene expression in patients with asthma indicate that GSDMA is a key factor in the disease pathogenesis (28) (**Table 1**). Hence, GSDMA is crucial in the immune response of the body to disease. Moreover, the interplay between DNA methylation and GSDMA expression plays a crucial role in an individual’s predisposition to asthma (29). Lower DNA methylation at promoter regions of GSDMA in peripheral blood cells of asthmatic individuals and in lymphoblastoid cell lines correspond to asthma-predisposing alleles (29).

GSDMB is highly expressed in the bronchial epithelium of asthmatic human lungs. In addition, it induces TGF-β1 expression (30). Several studies have shown that genetic variation in *GSDMB* is associated with asthma susceptibility and asthma-related phenotypes (**Table 1**), such as IgE11 and a change in FEV1 in response to albuterol (105, 120, 123–126). Positive as well as negative correlations have been observed between rs7216389 of *GSDMB* and asthma; a meta-analysis found moderate evidence of a correlation between *GSDMB* rs7216389 variants and asthma (127). GSDMB-regulated genes, cytokines, and chemokines, such as TGF-β1, MMP-9, 5-LO, cysteinyl leukotrienes (LTC4/D4/E4), HSP60/70, and CXCLs, contribute significantly to airway inflammation and remodeling in asthma (2). However, additional studies are required to determine whether GSDMB can be cleaved by inflammatory caspases in asthma. Similar to GSDMB, ORM1-like 3 in 17q is associated with asthma (128, 129). Thus, these 2 genes may be co-regulated, as their transcript levels appear to be connected (130). Larger, more comprehensive studies are needed to provide robust evidence of stratification, identify the interaction between genes and gene-environment interactions, and identify the factors important in asthma.

### Non-Syndromic Hearing Impairment

In 1998, *GSDME* (*DFNA5*) mutation was first identified as a cause of a specific form of progressive, non-syndromic and autosomal dominant hearing loss in a Dutch family (121). Later, many other families suffering from hearing loss were also found to have *GSDME* (*DFNA5*) mutations (**Table 1**) (31). Although these mutations differed at the DNA level, each skipped exon 8 of the *GSDME* (*DFNA5*) mRNA transcript, resulting in a frameshift mutation and premature truncation of the protein with cytotoxic activity (121, 131, 132). The mutation in exon 6 of *GSDME* (*DFNA5*) is not specific for hearing loss (HL). In fact, it is present in family members with normal hearing (133). These findings were supported by a study of *Gsdme* (*Dfna5*)-knockout mice, which did not display HL and suggest that GSDME (DFNA5)-associated HL is an activating and a gain-of-function mutation. The effect is associated with the reported apoptotic inducing capacity of GSDME (DFNA5), in which an increase in apoptosis leads to HL by inducing the death of cells that are crucial for hearing, such as cochlear hair cells (111). Similar to *GSDME* (*DFNA5*), mutations in *PJVK* are associated with HL in both humans and mice (20, 32, 65). Unlike the gain-of-function mutation of *GSDME* (*DFNA5*), all known mutations in *PJVK* are associated with autosomal recessive on syndromic sensorineural HL with or without cochlear dysfunction (32, 134–136). In mice, functional PJVK is necessary to enable existing peroxisomes to proliferate and protect the cochlear sensory hair cells and auditory neurons from noise-induced generation of reactive oxidative species (115). Interestingly, PJVK translocates to the membrane of peroxisomes in the inner hair cells and directly binds to the autophagy protein LC3B to induce autophagy-mediated removal of damaged peroxisomes (pexophagy) following oxidative stress caused by noise overexposure. Knockout of PJVK protects mice from peroxisomal dysfunction and sound-induced pexophagy (116). These results suggest that PJVK-induced pexophagy plays a crucial role in noise-induced peroxisome proliferation. Furthermore, these findings regard defective pexophagy as a cause of noise-induced hearing loss.

### Autoimmune- and Inflammation-Driven Diseases

Autoimmune diseases constitute a large group of diseases characterized by a functionally abnormal immune system. Aberrant activation of the immune system results in the production of antibodies against one own cells and tissues, known as autoimmunity. Several studies have implicated GSDMs in autoimmune and inflammation-driven diseases. GSDMA mutants have been linked to limited cutaneous system sclerosis (33, 34) and inflammatory bowel disease (**Table 1**) (35). The results of transethnic meta-analysis of genome-wide associated studies involving Japanese and European populations with a total of 4,436 cases and 14,751 controls revealed that a missense mutation in *GSDMA* (rs3894194) is associated with system sclerosis. The rs3894194 was strongly associated with GSDMB and ORM1-like 3 expression and the enhancer activity and enrichment of histone markers (33). RNA sequencing and genome-wide genotyping of monocyte-derived macrophages from patients with system sclerosis revealed that the *GSDMA* rs3894194 risk variant contributed to several inflammatory pathways and system sclerosis susceptibility, characterized by upregulation of glycolysis, hypoxia, and mTOR signaling and downregulation of the IFN-γ response pathway (137). In contrast to GSDMA, reduced expression of GSDMB increased the susceptibility to inflammatory bowel disease (35). However, another study pointed out that upregulated GSDMB in patients with sepsis and Crohn’s disease promoted caspase-4 activity and GSDMD cleavage (35), revealing a GSDMB-regulated novel regulatory mechanism for GSDMD-induced pyroptosis in inflammatory diseases.

Aberrant inflammasome activation and GSDMD-dependent pyroptosis are important pathogenic mechanisms underlying immune-related and inflammatory diseases, including atherosclerosis, type 2 diabetes mellitus, bullous pemphigoid, and cryopyrin-associated periodic syndromes (**Table 1**) (36–39). GSDMD-mediated pyroptosis plays a crucial role in the pathogenesis of familial Mediterranean fever in mouse models (138). Infection of familial Mediterranean fever knock-in macrophages with *Clostridium difficile* resulted in the expression of a chimeric familial Mediterranean fever-associated Mefv (V726A) pyrin-induced pyroptosis and GSDMD-mediated IL-1BIL-1B secretion *in vitro*. Importantly, GSDMD deletion protected mice from tissue damage. This characterized the autoinflammatory disease model *in vivo*, highlighting a potential strategy of GSDMD inhibition in inflammasome-driven diseases therapy. Unlike other GSDMs, DFNB59 lacks the cleavable linker domain. Thus, no study has reported that PJVK can form pores in the plasma membranes. Whether PJVK can be activated *via* inflammatory caspases and mediate pyroptosis requires further analysis.

### Sepsis and Septic Shock

Sepsis, as defined by the Third International Consensus Definitions for Sepsis and Septic Shock guidelines, is a life-threatening organ dysfunction caused by a dysregulated host response to infection (139). Septic shock is defined as severe sepsis, in which particularly profound circulatory, cellular, and metabolic abnormalities are associated with a greater risk of mortality than with sepsis alone. LPS, the major structural element of the outer membrane of gram-negative bacteria, triggers strong immune responses. In addition, LPS has been intensively implicated in the pathology of sepsis. Excessive LPS resulting from uncontrolled infection induces both sepsis and septic shock (140). Early studies demonstrated that in innate immunity, extracellular LPS is recognized by Toll-like receptor 4 (TLR4) to stimulate cytokine transcription. *Tlr4* ^-/–^deficient mice are resistant to endotoxic shock (141). Priming of the mice with TLR3 ligand to upregulate caspase-11 expression can bypass the requirement for TLR4 (84). Thus, the role of TLR4 in mouse endotoxic shock is largely limited to stimulation of caspase-11 expression. The re-defined role of TLR4 provides a plausible explanation for the failure of using the TLR4 antagonist Eritoran to treat patients with sepsis.

An important study conducted by Kayagaki et al. (25) revealed the crucial role of pro-inflammatory caspase-11 in caspase-1 activation and IL1b production in macrophages infected with *E. coli*, *Citrobacter rodentium*, or *Vibrio cholerae*. Following caspase 11 and caspase 1 gene manipulation in C57BL/6 mice, they provided evidence that caspase-11 (also known as human caspase-4) was critical for downstream caspase-1 activation. Their *in vivo* data also indicated that caspase-11 rather than caspase-1 is the critical effector of deleterious inflammatory responses in LPS-induced endothemia. Therefore, targeting human caspase-4 and caspase-5 (caspase 11 in mouse) may be more effective than inhibiting caspase-1 in patients with sepsis (25). *In vivo*, *Gsdmd^–/–^* and *Casp11^–/–^* mice show an increased susceptibility to infections with *Salmonella enterica* subsp. *enterica* serovar*, Typhimurium*, *SifA*, or *Brucella abortus* (142). In contrast, *Gsdmd^–/–^* mice were less susceptible to infection with *Francisella novicida* than *Casp1^–/–^* or *Aim2^–/–^* mice (143). Blood assays revealed that when primed with the TLR3 agonist poly(I:C), LPS-induced coagulation factor III (F3) release and lethal coagulation in mice suffering from sepsis required CASP11 but not CASP1-mediated GSDMD activation (69). Animal death caused by sepsis in response to intravenous injection of PAMP from *E. coli* was attenuated in mice with *Casp1*, *Casp1/11*, and *Gsdmd* depletion but not in *Tlr4^–/–^*, *Casp11^–/–^* mice or mice lacking receptors for IL-1B (*Il1r* ^–/–^) and IL-18 (*Il18r* ^–/–^). There is also evidence that intraperitoneal injection of necrosulfonamide, an inhibitor of the necroptosis effector mixed lineage kinase domain like pseudokinase, acts as a chemical inhibitor of GSDMD, thereby protecting mice from LPS-induced sepsis compared to controls (144). Thus, the critical role of intracellular LPS-caspase11/4/5-GSDMD axis-induced proptosis in sepsis provides a new avenue for the development of anti-septic therapeutics and merits further investigation (**Table 1**).

### Other Diseases

Additionally, NLRP3/caspase-1/GSDMD activation and subsequent pyroptosis are associated with the pathogenesis of brain injury in a middle cerebral artery occlusion/reperfusion rat model (145). GSDMD activation and pytoptosis are induced in the brain tissues of mice after ischemia/reperfusion damage. Furthermore, GSDMD-NT can bind to membrane lipids and exhibit membrane-disrupting cytotoxicity in primary-cultured microglia, depending on the Glu15 and Leu156 amino acid sites. Inhibition of GSDMD and its upstream modulators, such as MCC950, LDC7559, and necrosulfonamide, with chemical compounds can attenuate the progression of diseases such as hepatic ischemia-reperfusion injury in rat models (146) and LPS-induced septic death (48, 144). Serum-derived exosomes from hepatic ischemia-reperfusion injury can not only cross the blood-brain barrier but also contribute to hippocampal and cortical neuronal pyroptosis *via* NLRP3-caspase-1-GSDMD activation and IL-1BIL-1B and IL-18 release in rat models (146). The small molecular specific inhibitor of NLRP3 MCC950 inhibits caspase-1 and GSDMD activation and attenuates subsequent hippocampal and cortical neuronal injury in a hepatic ischemia-reperfusion model of rats.

## Concluding Remarks and Future Perspectives

Reports on GSDMD as a key effector of pyroptosis have improved the understanding of the GSDM family as an important executor of cell death. Many studies have described the role of gasdermins in regulating various physiological and pathological processes, including cell differentiation, cell death, coagulation, inflammation, and tumorigenesis. Studies of the structure and mutagenesis of full-length gasdermins revealed the mechanism of self-inhibition and membrane insertion. The GSDM-NT domains released following cleavage of GSDMA, GSDMB (or full-length GSDMB), GSDMC, GSDMD, or GSDME can form membrane pores, thereby acting as executors of pyroptosis. Specific gasdermin inhibitors have only recently been identified and require further analysis. Targeting the activity and function of GSMDs provides a potential strategy for treating diseases, particularly infections and inflammatory conditions. Therefore, studies are needed to evaluate the distinct resources of gasdermins in different cellular processes and their biological and pathological effects, identify shear molecules responsible for PJVK, discovering other functions besides cell lysis and pyroptosis, recognizing other regulators of GSDMs in cell death, and developing a GSDM-dependent strategy for disease treatment.

## Author Contributions

Conceptualization, RC and JZ. Writing—original draft, RC. Writing—review and editing, DT, RK, YZ, YH, and JZ. All authors contributed to the article and approved the submitted version.

## Funding

The manuscript was supported by grants from the National Natural Sciences Foundation of Hunan province (#2019JJ30041), National Natural Sciences Foundation of China (#82070613), and Innovation-Driven Project of Central South University (#2020CX044).

## Conflict of Interest

The authors declare that the research was conducted in the absence of any commercial or financial relationships that could be construed as a potential conflict of interest.

## Publisher’s Note

All claims expressed in this article are solely those of the authors and do not necessarily represent those of their affiliated organizations, or those of the publisher, the editors and the reviewers. Any product that may be evaluated in this article, or claim that may be made by its manufacturer, is not guaranteed or endorsed by the publisher.

## Abbreviations

GSDM, gasdermin; GSDMA, gasdermin A; GSDMB, gasdermin B; GSDMC, gasdermin C; GSDMD, gasdermin D; DFNA5, deafness autosomal dominant 5; Rim3, recombination-induced mutation 3; CASP1, chloroquine down-regulated caspase-1; PJVK, pejvakin; GSDM-NT, GSDM N-terminal; GSDM-CT, GSDM C-terminal; LPS, lipopolysaccharide; NET, neutrophil extracellular trap; HL, hearing loss; FMF, familial Mediterranean fever; TLR4, Toll-like receptor 4.
